# Food Anticipatory Activity on Circadian Time Scales Is Not Dependent on Central Serotonin: Evidence From Tryptophan Hydroxylase-2 and Serotonin Transporter Knockout Mice

**DOI:** 10.3389/fnmol.2020.534238

**Published:** 2020-09-11

**Authors:** Christian M. Gallardo, Camille S. Martin, Andrew D. Steele

**Affiliations:** ^1^Division of Biology, California Institute of Technology, Pasadena, CA, United States; ^2^Department of Biological Sciences, California State Polytechnic University Pomona, Pomona, CA, United States

**Keywords:** circadian rhythm, food entrainment, food anticipatory activity, Slc6a4, tryptophan hydroxylase-2

## Abstract

A number of studies implicate biogenic amines in regulating circadian rhythms. In particular, dopamine and serotonin influence the entrainment of circadian rhythms to daily food availability. To study circadian entrainment to feeding, food availability is typically restricted to a short period within the light cycle daily. This results in a notable increase in pre-meal activity, termed “food anticipatory activity” (FAA), which typically develops within about 1 week of scheduled feeding. Several studies have implicated serotonin as a negative regulator of FAA: (1) aged rats treated with serotonin 5-HT2 and 3 receptor antagonists showed enhanced FAA, (2) mice lacking for the 2C serotonin receptor demonstrate enhanced FAA, and (3) pharmacologically increased serotonin levels suppressed FAA while decreased serotonin levels enhanced FAA in mice. We sought to confirm and extend these findings using genetic models with impairments in central serotonin production or re-uptake, but were surprised to find that both *serotonin transporter* (*Slc6a4*) and *tryptophan hydroxylase-2* knockout mice demonstrated a normal behavioral response to timed, calorie restricted feeding. Our data suggest that FAA is largely independent of central serotonin and/or serotonin reuptake and that serotonin may not be a robust negative regulator of FAA.

## Introduction

Biological rhythms influence nearly all cellular, physiological, and behavioral processes: from metabolic rate (Oklejewicz et al., 1997) to cancer stem cell proliferation (Puram et al., 2016), xenobiotic metabolism (DeBruyne et al., 2014), and glucose homeostasis (Versteeg et al., 2015). The high conservation of circadian rhythms suggests the importance of keeping cellular processes coordinated with the external environment (Bell-Pedersen et al., 2005). Much is known about how light influences circadian rhythms of mammals by regulating the activity of neurons in the suprachiasmatic nucleus (SCN) (Mohawk et al., 2012). Neurons within the SCN serve as a molecular clock, orchestrating the release of hormones and other physiological processes regulated by the hypothalamus (Welsh et al., 2010). In contrast to light, comparatively little is known about how other environmental cues, such as feeding, tune circadian rhythms (Steele and Mistlberger, 2015).

Given the prevalence of obesity and other eating disorders, such as anorexia nervosa and binge eating, it is vital to determine the mechanism(s) by which circadian rhythms control appetite and determine how feeding influences circadian processes (reviewed in Froy, 2010). In rodent model systems, food entrainment is typically studied by either calorically or temporally limiting food access (Mistlberger, 2011). Once mice have been maintained on a feeding schedule for about 1 week they will develop food anticipatory activity (FAA), which is a marked increase in arousal and physical activity in the 2–3 h preceding scheduled mealtime (Mistlberger, 2011). The underlying neuronal systems and/or circuitry responsible for mediating FAA are hotly contested, with very few studies showing reproducible effects of mutations or lesions (Davidson, 2009; Gunapala et al., 2011; Pendergast and Yamazaki, 2018). At present, nuclei as diverse as the cerebellum (Mendoza et al., 2010) and striatum (Gallardo et al., 2014a) have been implicated in promoting FAA. The only area of consistent agreement is that the SCN is not required for FAA (Davidson, 2009), although it might modulate the amplitude of food rhythms (Acosta-Galvan et al., 2011).

Serotonergic neurons heavily innervate the SCN, and serotonin (5-HT) has well-documented effects on both circadian rhythms (Morin and Blanchard, 1991; Pickard and Rea, 1997; Mistlberger et al., 1998; Versteeg et al., 2015) and feeding behavior (Tecott et al., 1995; Xu et al., 2008; Wu et al., 2012; Voigt and Fink, 2015), making it a very likely candidate for mediating FAA. For example, mice lacking 5-HT2C receptor show hyperphagia, hyperactivity, and increased susceptibility to obesity (Tecott et al., 1995). Further studies using conditional knockout (KO) of 5-HT2C receptors indicate that proopiomelanocortin (POMC) neurons in the arcuate nucleus are responsible for most of the effects of serotonin on feeding and activity levels (Xu et al., 2008). The 5-HT2C receptor KO mice showed an enhanced FAA in response to time-restricted feeding, suggesting that 5-HT negatively regulates FAA (Hsu et al., 2010). This notion was supported by an earlier study of 5-HT by Shibata and colleagues, who used 5-HT receptor antagonists (5-HT2 receptor antagonists, mianserin and ritanserin, and a 5-HT3 receptor antagonist, Y25130) to rescue FAA in aged rats in a short-term time restricted feeding study (Shibata et al., 1995). More recently, another pharmacological study described 5-HT as a negative regulator of FAA and a possible “resetting” function of 5-HT of food entrained activity rhythms (Rozenblit-Susan et al., 2016). Using an inhibitor for the rate limiting enzyme for 5-HT production, tryptophan hydroxylase (Tph), Rozenblit-Susan et al. (2016) observed a free running component of FAA (in constant darkness) and that treatment with a selective serotonin reuptake inhibitor (SSRI) decreased the amplitude of FAA.

Based on these results, we sought to extend investigations of 5-HT as a suppressor of FAA in order to delineate the nuclei critical for circadian entrainment to scheduled feeding. As *Sert* (*Slc6a4*) KO mice have increased levels of synaptic 5-HT due to lack of reuptake (Bengel et al., 1998), we expected that homozygous mutant mice would have diminished FAA. In contrast, deletion of *Tph2*, which is responsible for the bulk of central 5-HT production (Walther et al., 2003; Zhang et al., 2004), should also enhance FAA. Here we report the surprising result that both *Sert* KO and *Tph2* KO mice showed FAA comparable to controls in response to timed, calorie restricted (CR) feeding.

## Materials and Methods

### Mouse Strains, Husbandry, and Serotonin Measurement

The animal study was reviewed by the California Institute of Technology’s Animal Care and Use Committee. Mice were maintained in static microisolator cages in the following environmental conditions: 13:11 light:dark cycle, temperatures ranged between 21 and 23°C, and humidity ranged between 45 and 65%. By convention for non-12:12 L:D cycle, ZT 12 was designated as the commencement of lights-off. The cages contained sani-chip bedding and a cotton nestlet. Mice were fedRodent Chow 5001 (LabDiet); water was provided *ad libitum* for the duration of the study.

*Sert* KO mice (Bengel et al., 1998) were purchased from Jackson labs (stock number 008355) and *Tph2* KO (Wu et al., 2012) on a C57BL/6J background were obtained from the laboratory of Michael Clarke (University of Washington, Seattle). The *Tph2* targeting construct was prepared by flanking the first exon with loxP sites (Wu et al., 2012) and the floxed exon was germ-line excised after breeding of Tph2 floxed mice with Cre-deleter B6.129S4-*Meox2^TM1(cre)Sor)^*/J mice. To genotype mice, DNA was obtained from tail clippings from 2 week old mice and then digested with proteinase K. DNA was purified using an isopropanol precipitation and was amplified by genomic PCR. Heterozygous mutant mice were intercrossed to produce +/+, +/-, and -/- progeny used in experiments. For genotyping the *Sert* locus, the following primers were used: mutant reverse GCCAGAGGCCACTTGTGTAG, common AATGGTGAGGAGTGGTGGAG and WT reverse CCTAGATACCAGGCCCACAA. *Sert* wild-type mice amplify a 318 bp product, homozygous mutants amplify at 210 bp product, while heterozygotes have both bands. For PCR genotyping the *Tph2* locus, primers ACCAATGTTAACATATACAGTCTTGC (fw) and CAATTTGACAGGCATAGACAAG (rev) were used to detect a 213 bp WT band, while ACCAATGTTAA CATATACAGTCTTGC (fw) and CCTTTGCAAGAACT GTAAC (rev) were used to detect a 418 bp band for the KO allele.

For *Sert*, *n* = 5 controls were +/- and *n* = 4 were +/+; these groups were combined as a single “control” group because there was no differences in FAA between +/+ and +/- mice. For *Tph2*, *n* = 12 controls were +/+ and *n* = 3 were +/-. We combined +/ + and +/- mice in one control group because there was no evidence for an effect of gene dosage on behavior. Both males and females were used in these studies. For *Sert*, *n* = 7 control male, *n* = 2 control female, *n* = 5 control KO male, and *n* = 4 KO female. For *Tph2*, *n* = 8 control male, *n* = 7 control female, *n* = 6 -/- male and *n* = 6 -/- female.

To measure central 5-HT, 0.5 mm diameter 2 mm thick tissue punches were taken using “NIH Style Neuro Punches” (Fine Science Tools) from the dorsal raphe and ventral midbrain and immediately frozen in liquid nitrogen. Frozen samples were sent to the Neurochemistry Core Laboratory at the Center for Molecular Neuroscience Research of Vanderbilt University (Nashville, TN), where HPLC-coupled with electrochemical detection was used to measure 5-HT content relative to total protein, as determined by BCA protein assay (Thermo Scientific). Tissues were homogenized, using a handheld sonic tissue dismembrator, in 100–50 μl of 0.1 M TCA containing 0.01 M sodium acetate, 0.1 mM EDTA, and 10.5% methanol (pH 3.8). The samples were centrifuged in a microcentrifuge at 10,000 g for 20 min. The supernatant was removed for HPLC-ECD analysis. HPLC was performed using a Kinetix 2.6 μm C18 column (4.6 × 100 mm, Phenomenex, Torrance, CA, United States). The same buffer used for tissue homogenization is used as the HPLC mobile phase.

### Calorie Restriction Studies and Measurements of Home Cage Behavior

Mice were single housed (for the duration of the study) for at least 3 days prior to measuring food intake, which was measured across 3–4 days to calculate an average daily food intake for each group of mice. To test for FAA, mice were then allocated 60% of the group average *ad libitum* intake daily at ZT 8. Mice typically consume their entire meal in less than 2 h once they have been on a CR feeding schedule for 1 week. All mice were 9–10 weeks old at the start of CR. Body weight measurements were conducted weekly, beginning 1 week prior to initiating 60% CR (“day -7”), and we maintain weight loss between 10 and 15% for all groups of mice by adjusting CR values by 0.1 grams per day as needed. Experimenters were blind to the genotypes of the mice during the behavioral study.

Mice were maintained in single housing and were video recorded once per week for 24 h. Food was presented at the start of the video recording. Video recordings were analyzed by computer vision software, HomeCageScan 3.0 (Clever Systems, Inc.), which quantified home cage behaviors as previously described (Steele et al., 2007). For the purposes of our analysis, we only examined data reflecting the highest arousal states: rearing, jumping, walking, and hanging for each hour of recording. Other behaviors scored by HCS, including sleep, twitch (movement during sleep), remain low, pause, groom, turn, drink, food bin entry, stretch, sniff, unknown behavior and no data were ignored. Food bin entry data is not included in our definition of “high activity” behaviors but generally is very similar to rearing data. Video data was not used when the amount of no data and unknown behaviors exceeded 5% of total time. Examination of the details of home cage behaviors can be superior to using running wheels to measure FAA, as discussed in Gunapala et al. (2011).

### Data Analysis

We define FAA as the amount of normalized high activity behaviors occurring in the 3 h preceding scheduled mealtime. This value is computed by dividing the seconds of hanging, jumping, walking and rearing in the 3 h prior to scheduled feeding by the total amount of hanging, jumping, walking and rearing in the entire 24 h video recording, expressed as a fraction of total. For statistical analysis of behavioral data, we used a mixed-effects analysis and Sidak’s multiple comparisons test to examine pairwise differences. We used mixed-effects model because it accommodates missing data (whereas 2-way ANOVA cannot be used for datasets with missing values). GraphPad Prism version 8 was used for all statistical tests and graphing. Graphs display means ± SEM with all sample values shown as a scatter plot; sample sizes are indicated in the Figure legends.

## Results

**FIGURE 1:**
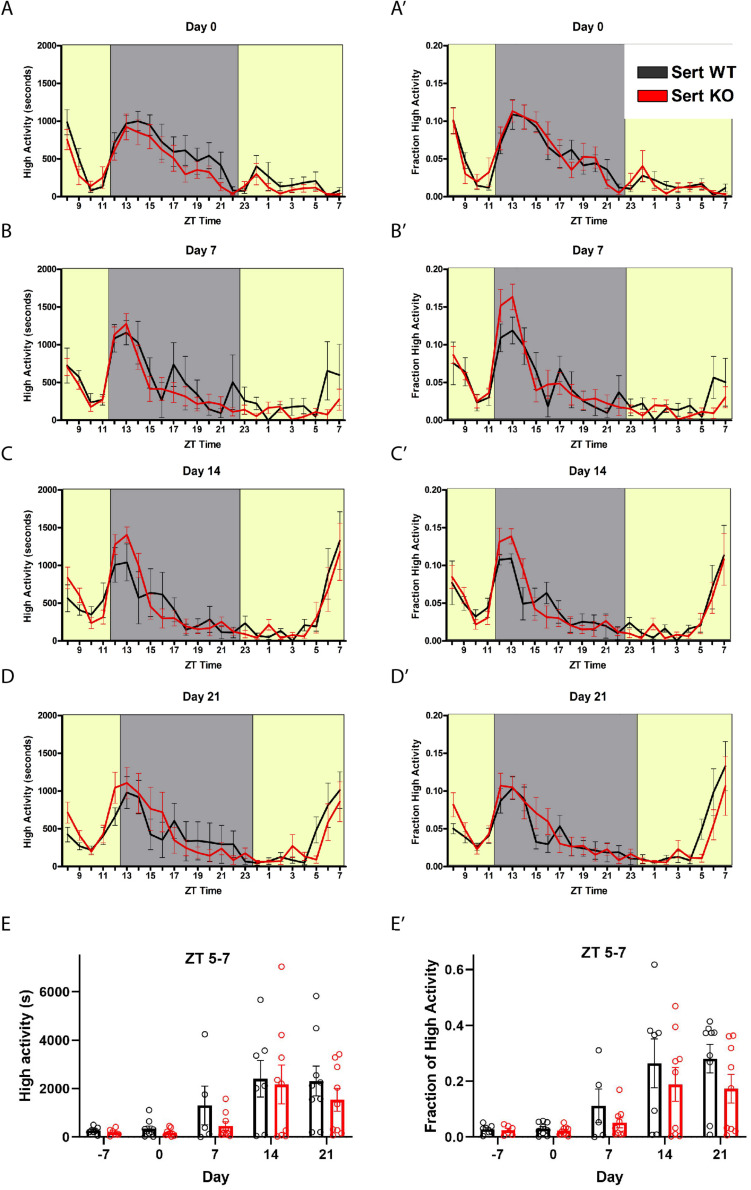
Food anticipatory behavior in serotonin transporter knockout mice. **(A)** Mean ± SEM seconds of high activity behaviors (hanging, jumping, rearing, and walking) on the first day (“day 0”) of scheduled CR feeding. The yellow area indicated lights on and the gray area indicates lights off. **(A’)** Normalized mean ± SEM high activity of data shown in **(A)**. **(B)** Mean ± SEM seconds of high activity behaviors after 7 days of timed CR feeding. **(B’)** Normalized mean ± SEM high activity of data shown in **(B)**. **(C)** Mean ± SEM seconds of high activity behaviors after 14 days of timed CR feeding. **(C’)** Normalized mean ± SEM high activity of data shown in **(C)**. **(D)** Mean ± SEM seconds of high activity behaviors after 21 days of timed CR feeding. **(D’)** Normalized mean ± SEM high activity of data shown in **(D)**. **(E)** Mean ± SEM seconds of high activity data in the 3 h preceding scheduled feeding across the experiment (sum of ZT 5, 6, and 7). **(E’)** Mean ± SEM normalized high activity data shown in **(E)** in the 3 h preceding scheduled feeding across the experiment. *n* = 5–9 controls and *n* = 6–9 KO per time point.

We tested the behavioral responses of *Sert* KO mice to timed feeding presented at ZT8. We gave *Sert* KO and controls (denoted as WT but were a mixture of + / + and +/-) 60% of their *ad libitum* food intake (termed “60% CR”) at ZT8 daily for 21 days. Upon initiation of timed CR at day 0, both *Sert* KO and age-matched control mice showed a similar amount of time (in seconds) engaged in high activity behaviors, that is, hanging, jumping, rearing, and walking, upon being placed in a new cage at ZT 8 and then again when the lights turn off at ZT 12 (Figure 1A). When data were normalized by dividing the seconds of high activity in each hr by the total high activity over 24 h, the normalized waveforms of high activity were similar between *Sert* KO and control mice (Figure 1A’). By day 7 of timed, CR feeding, the control and Sert KO mice began to demonstrate pre-meal activity (Figures 1B,B’). There were no difference in pre-meal activity on days 14 or day 21 both in terms of seconds and normalized high activity (Figures 1C,C’,D,D’). We plotted the amount of high activity in seconds (Figure 1E) and normalized (Figure 1E’) in the 3 h preceding scheduled mealtime, our working definition of FAA, across the duration of the experiment. For the seconds of high activity data, mixed-effects analysis revealed a statistically significant effect of time [*P* < 0.0001, *F*(4, 52) = 11.55], but no effect of either genotype [*P* = 0.415; *F*(1, 17) = 0.6989] or the interaction of time and genotype [*P* = 0.864, *F*(4, 52) = 0.3186]. Examination of pairwise differences using Sidak’s multiple comparisons test did not reveal significant differences between groups at any timepoints. For normalized FAA data, the mixed-effects analysis confirmed a significant effect of time [*P* < 0.0001, *F*(2.047, 26.61) = 15.37], but no effect of either genotype [*P* = 0.278; *F*(1, 17) = 1.255] or the interaction of time and genotype [*P* = 0.649, *F*(4, 52) = 0.6217]. Examination of pairwise differences using Sidak’s multiple comparisons test did not reveal significant differences between groups at any timepoints. In summary, there were no significant differences in pre-meal activity, either in seconds or normalized, between *Sert* KO and control mice throughout the experiment.

**FIGURE 2:**
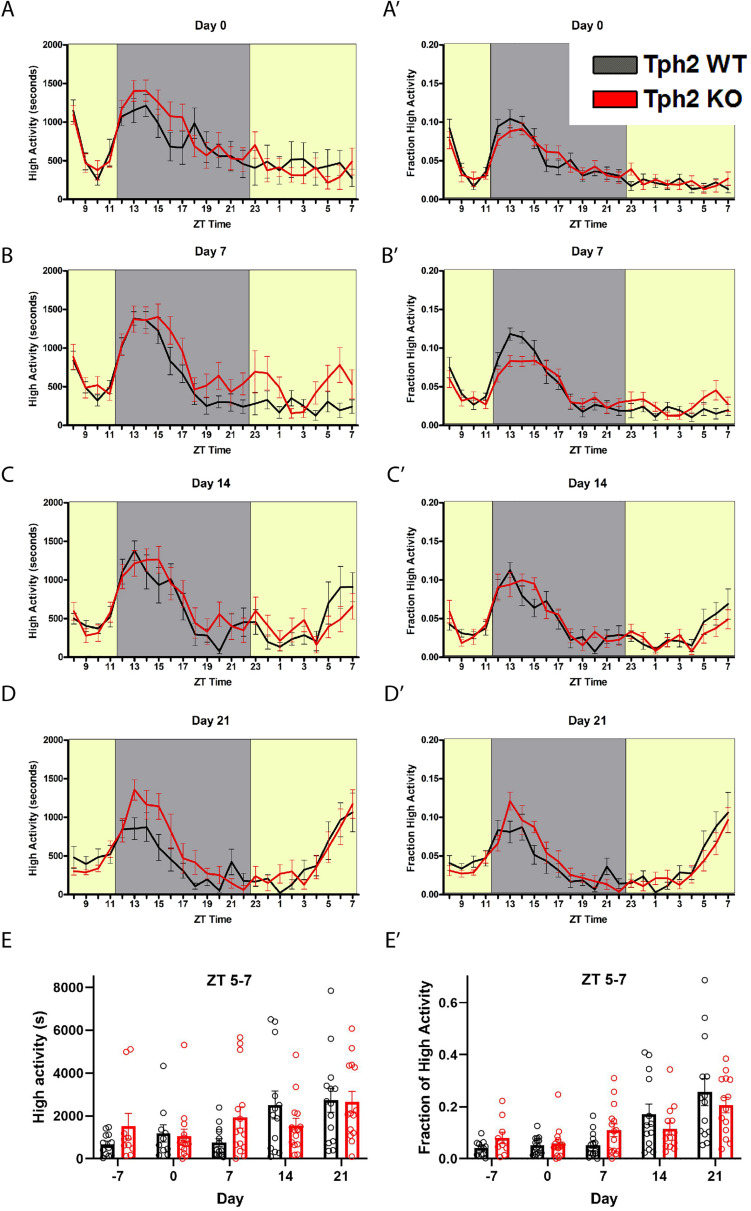
Food anticipatory behavior in Tryptophan hydroxylase 2 knockout mice. **(A)** Mean ± SEM seconds of high activity behaviors (hanging, jumping, rearing, and walking) on the first day (“day 0”) of scheduled CR feeding. **(A’)** Normalized mean ± SEM high activity of data shown in **(A)**. **(B)** Mean ± SEM seconds of high activity behaviors after 7 days of timed CR feeding. **(B’)** Normalized mean ± SEM high activity of data shown in **(B)**. **(C)** Mean ± SEM seconds of high activity behaviors after 14 days of timed CR feeding. **(C’)** Normalized mean ± SEM high activity of data shown in **(C)**. **(D)** Mean ± SEM seconds of high activity behaviors after 21 days of timed CR feeding. **(D’)** Normalized mean ± SEM high activity of data shown in **(D)**. **(E)** Mean ± SEM seconds of high activity data in the 3 h preceding scheduled feeding across the experiment. **(E’)** Mean ± SEM normalized high activity data in the 3 h preceding scheduled feeding across the experiment. *n* = 12–15 WT and *n* = 10–16 KO per time point.

Given that *Sert* deletion did not appear to alter FAA, we next tested mice deleted for *Tph2*, which is responsible for the bulk of central 5-HT production. Measurements of 5-HT (ng/mg total protein) from tissue punches (*n* = 2 per group) taken from the dorsal raphe nucleus were reduced to 50.2% of control for +/- , and 2.3% for -/- *Tph2* mutant mice (Table 1). We also measured 5-HT levels from tissue punches (*n* = 2 per group) taken from the ventral midbrain and observed that ± mice had 72.2% of control serotonin levels while the *Tph2* -/- mice had only 1.81% of control values (Table 1). Consistent with previous results (e.g., Alenina et al., 2009), the starting body weight of the Tph2 KO was approximately 5 grams less than that of controls: mean weight for WT was 24.2 (± 3.8 *SD*) and for KO 19.1 ± 3.3 (*SD*) grams (*P* < 0.0001, Sidak’s multiple comparisons test). Next, we tested the behavioral responses of mice deleted for *Tph2* and +/- and + / + controls to 60% CR timed feeding presented at ZT 8. Given the low levels of central 5-HT in *Tph2* KO mice, we expected that the mutant mice would have increased FAA. Upon initiating CR (Day 0), the activity waveforms of age-matched *Tph2* KO and control (denoted as WT but were a mixture of + / + and ±) mice were similar, both groups showed a strong arousal from being placed in a new home cage and a large increase in activity upon the onset of the dark phase (Figure 2A). Normalized high activity data revealed similar waveforms for *Tph2* KO and control mice at day 0 (Figure 2A’). The *Tph2* KO group showed a trend toward increased activity (in seconds) preceding mealtime at day 7 of CR, but this increase was not statistically significant (Figure 2B), nor was the normalized high activity value significantly increased (Figure 2B’). At days 14 and 21, both groups of mice showed notable increases in activity in the 2–3 h preceding scheduled mealtime (Figures 2C,D). We plotted the amount of high activity (in seconds, Figure 2E) and normalized (Figure 2E’) in the 3 h preceding scheduled mealtime across the duration of the experiment and there were no significant differences between *Tph2* KO and control values. For the seconds of high activity data, mixed-effects analysis revealed a statistically significant effect of time [*P* < 0.0036, *F*(2.801, 62.32) = 5.169], but no effect of either genotype [*P* = 0.565; *F*(1, 32) = 0.3387] or the interaction of time and genotype [*P* = 0.120, *F*(4, 89) = 1.886]. Examination of pairwise differences using Sidak’s multiple comparisons test did not reveal significant differences between groups at any time points. For normalized FAA data, the mixed-effects analysis confirmed a significant effect of time [*P* < 0.0001, *F*(2.028, 48.67) = 16.37], but no effect of either genotype [*P* = 0.954; *F*(1, 32) = 0.003401] or the interaction of time and genotype [*P* = 0.116, *F*(4, 96) = 1.906]. Examination of pairwise differences using Sidak’s multiple comparisons test did not reveal significant differences between groups at any time points.

**TABLE 1:** Serotonin levels in individual mice from tissue punches taken from the ventral midbrain and the dorsal raphe nucleus (ng/mg of tissue).

**Tph2**	**+/+**	**+/+**	**+/−**	**+/−**	**−/−**	**−/−**
Ventral midbrain	5.93	8.46	2.97	7.35	0.13	0.55
Dorsal Raphe	5.58	9.39	3.86	3.68	0.21	0.14

## Discussion

Despite at least three published studies implicating 5-HT as a negative regulator of circadian entrainment to feeding, we observed no evidence for the contribution of 5-HT signaling using genetic manipulations. Mice with increased extracellular 5-HT (Mathews et al., 2004; Fox et al., 2007), *Sert* mutants, showed similar FAA to controls. *Tph2* KO mice, which based on our measurements have a 40–50-fold reduction in 5-HT in the midbrain and hindbrain, had a similar amount of FAA as controls. It is also worth noting that *Tph2* KO mice have been generated by several labs and yielded a wealth of behavioral and other phenotypes (Savelieva et al., 2008; Alenina et al., 2009; Narboux-Nême et al., 2012; Angoa-Pérez et al., 2014, 2015; Mosienko et al., 2015), so our failure to detect an effect on FAA cannot be attributed to compensatory effects of *Tph1* upregulation, for example. However, lifelong reduction (*Tph2* KO) or increases in brain serotonin (*Sert* KO) could lead to reestablishment of serotonergic system and that may blunt the effects seen by the acute depletion or enhancement of serotonin levels (Descarries and Riad, 2012). Experiments involving more acute manipulations could include examination of FAA in mice with post-natal, conditional deletion of *Tph2*, such as in the study of Whitney et al. (2016), who achieved nearly complete depletion of central 5-HT using adeno-associated viral vectors to deliver *Cre recombinase* to mice with loxP flanked *Tph2* alleles. Another approach would be to avoid deletion of *Tph2* altogether and focus on manipulating the activity of 5-HT neurons using chemogenetic activation or silencing methods in mice on scheduled feeding regimens.

There are several methodological differences between our study and the previous studies investigating the role of 5-HT in circadian entrainment to scheduled feeding. Firstly, all the prior studies of 5-HT and FAA (Shibata et al., 1995; Hsu et al., 2010; Rozenblit-Susan et al., 2016) measured activity using running wheels, which are known to enhance FAA (Flôres et al., 2016), whereas we measured high intensity activity behaviors occurring in the normal home cage. Possibly most importantly, the studies by Shibata et al. (1995) and Rozenblit-Susan et al. (2016) study used pharmacology, and SSRI medications have been noted to have pleiotropic effects (Isaac et al., 2013). Rozenblit-Susan et al. (2016) demonstrated that parachlorophenylalanine treatment, the model for 5-HT depletion, showed increased activity under LD conditions. During restricted feeding, this 5-HT depleted group showed activity with free running of the FAA in DD conditions only; there were no differences in FAA levels or onset during LD. Additionally, they demonstrated that under restricted feeding conditions, the fluvoxamine-treated group, which served as a model for prolonged exposure to 5-HT, lead to a reduced FAA during LD. Hsu et al. (2010) used a genetic model to assess serotonergic contributions to FAA, observing that 5-HT2C deletion enhances FAA and increased c-Fos RNA levels in several brain regions, including the nucleus accumbens. Moreover, it is also worth noting that the 2C receptor have intrinsic activity that is independent of 5-HT (Grotewiel and Sanders-Bush, 1999), such that deletion of the receptor and use of antagonists would be 5-HT-independent. The authors note that there is evidence that dopamine neurotransmission is enhanced in this KO model (Abdallah et al., 2009), which is of interest because several studies implicate the dopamine system in promoting FAA (Smit et al., 2013; Gallardo et al., 2014a; Michalik et al., 2015; Steele and Mistlberger, 2015). Thus, it is possible that the 5-HT2C mutant might have enhanced FAA due to heightened dopamine release thresholds. In line with this reasoning, chronic SSRI treatment lowers dopamine levels in the basal ganglia and this could be overcome by treatment with l-DOPA (Morelli et al., 2011), possibly explaining why SSRI treatment blunts FAA.

## Conclusion

We conclude based on our current evidence that 5-HT is unlikely to be a robust negative regulator of FAA in mice.

## Data Availability Statement

The datasets generated for this study are available on request to the corresponding author.

## Ethics Statement

The animal study was reviewed and approved by the California Institute of Technology Institutional Animal Care and Use Committee.

## Author Contributions

AS and CG designed the experiments. AS, CG, and CM performed the experiments. AS and CM analyzed the data. AS wrote the manuscript. All authors approved the final version.

## Conflict of Interest

The authors declare that the research was conducted in the absence of any commercial or financial relationships that could be construed as a potential conflict of interest.
